# Osteochondral Allograft Transplantation to the Capitellum: Technical Considerations of a Mega Osteochondritis Dissecans Technique

**DOI:** 10.1016/j.eats.2024.102997

**Published:** 2024-04-17

**Authors:** Jeffrey D. Hassebrock, David R. Diduch, A. Bobby Chhabra

**Affiliations:** a Department of Orthopedic Surgery, Mayo Clinic Arizona, Phoenix, Arizona, U.S.A.; b Department of Orthopedic Surgery, University of Virginia, Charlottesville, Virginia, U.S.A.

## Abstract

Osteochondritis dissecans of the elbow is a rare but debilitating pathology typically found in the adolescent repetitive overhead athlete. In the setting of unstable lesions, mechanical symptoms, or deteriorating function despite appropriate conservative management, surgical osteochondral allograft transplantation of the capitellum is a viable option for even large lesions (>10 mm), with minimal morbidity and good return of function. We describe a technique for performing a large osteochondral allograft transplantation of the capitellum.

Osteochondritis dissecans (OCD) of the elbow is a well-described pathology. Despite a lower incidence overall in terms of elbow pathologies, there is a higher prevalence among repetitive overhead athletes such as baseball players and gymnasts. Repetitive loading across the radial head into the capitellum is thought to cause microtrauma and eventual injury to the subchondral blood supply to the capitellum, resulting in an OCD lesion.^1^^,^^2^

Strategies exist for the treatment of this pathology and have ranged historically from nonoperative relative rest to surgical intervention.^2^ In patients with progressively worsening mechanical symptoms or pain or those who have failed appropriate conservative therapy, multiple surgical interventions have been described.^3^ Osteochondral autograft transplantation from the ipsilateral knee has been well described, with excellent results but a concerning donor site morbidity rate in some studies.^4^^,^^5^ This donor site morbidity had led some surgeons to pursue osteochondral allograft transplantation for the treatment of these lesions.^4^^,^^6^^,^^7^ Our described technique illustrates a surgical approach for large (>10 mm) osteochondral allografts for the treatment of symptomatic capitellar lesions.

## Surgical Technique

### Step 1: Preoperative Workup

**Fig 1:**
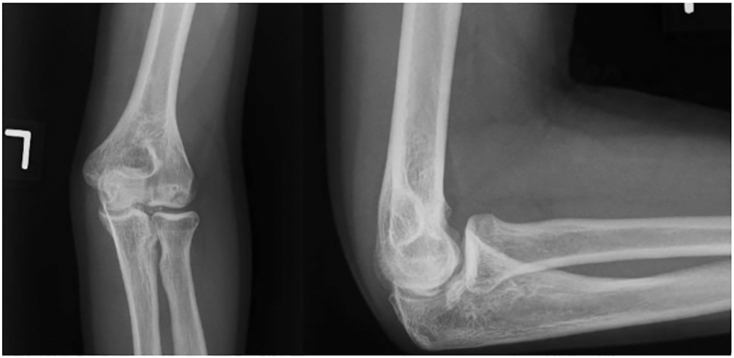
AP and lateral of left elbow showing an OCD lesion of the capitellum. (AP, anteroposterior; OCD, osteochondritis dissecans.)

**Fig 2:**
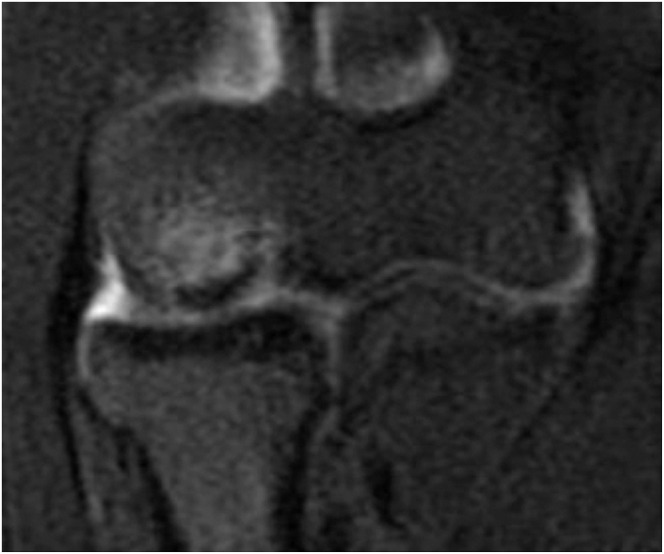
Coronal view of a T2 MRI sequence of left elbow showing a large (>14 mm) unstable capitellar OCD lesion. (MRI, magnetic resonance imaging; OCD, osteochondritis dissecans.)

All patients with symptomatic osteochondral lesions of the capitellum undergo a standard radiographic workup, which includes anteroposterior and lateral elbow radiographs of the symptomatic side as well as the contralateral asymptomatic elbow (Fig 1). Additional work-up includes magnetic resonance imaging without contrast, which enables characterization of the lesion as well as stability of the lesion itself (Fig 2).

In addition to imaging work-up, physical examination and history are crucially important. All patients undergo an evaluation of their sport, mechanism, and symptoms as well as a detailed examination assessing for crepitus, range of motion loss, and changes to these variables over time. After a 6-month trial of conservative management with symptomatic treatment and relative rest, patients with ongoing symptoms are evaluated for osteochondral surgical intervention.

Once indicated for surgical intervention, graft choice is discussed in detail with the patient. The gold standard for small osteochondral capitellar lesions has traditionally been described as ipsilateral distal femur autograft transplantation. Other options include autograft costochondral transplantation or alternate allografts. Risks and benefits of the graft choices are discussed in detail with the patient (Table 1). When the measured defect is large (>14 mm in any dimension), the benefits of decreasing autograft harvest morbidity are discussed with the patient, and allograft options are often selected. The following surgical technique describes distal femoral allograft osteochondral transplantation in the setting of a large defect (mega OCD).

**Table 1:** Advantages and Disadvantages of Lateral Femoral Condyle OCA to the Capitellum

Advantages	Disadvantages
No donor site morbidity.	Cost is substantial and insurance coverage for graft is uncommon.
Availability is more common than distal humerus allograft. Excellent radius of curvature match to capitellum.	Infectious disease risk present compared with autograft.

OCA, osteochondral allograft.

### Step 2: Surgical Positioning and Diagnostic Arthroscopy

**Fig 3:**
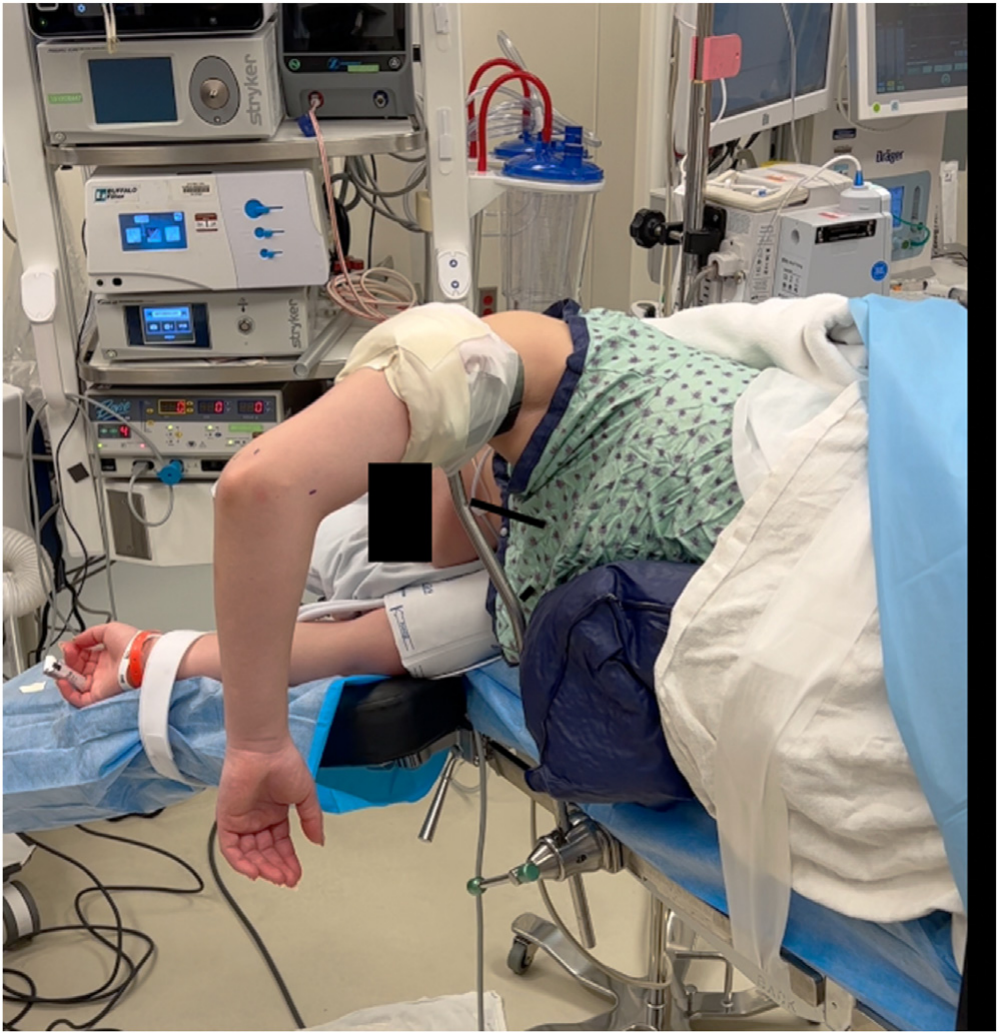
Standard right lateral decubitus positioning for left elbow diagnostic arthroscopy, anteromedial and posterior portals demarcated.

The patient is placed in the lateral decubitus position with the operative extremity exposed. The surgical preference of the senior author (A.B.C.) is to drape the operative extremity over a nonsterile arm holder and tape the extremity to the arm holder to prevent shifting during application of subsequent drapes for the arthroscopy portion of the case. The contralateral extremity and legs are well padded to prevent iatrogenic axillary or peroneal nerve injury. The operative extremity is prepped and draped in the usual sterile fashion and the arm is exsanguinated with a sterile tourniquet (HemaClear; OHK Medical Devices). Standard diagnostic elbow arthroscopy is performed using an anteromedial portal for viewing and an anterolateral portal for working in the anterior compartment. Additional posterior and accessory posterolateral portals are created to visualize posteriorly and to remove loose bodies (Fig 3).

It is the senior author’s preference to perform elbow arthroscopy in all of these cases, as the rate of concomitant loose bodies and inflamed synovium is high. After completion of the elbow arthroscopy, the portals are closed with nonabsorbable suture and a sterile dressing is applied. The drapes are removed and the patient is repositioned under the same anesthesia in the supine position with the operative extremity placed on an arm table.

### Step 3: Secondary Positioning and Approach

**Fig 4:**
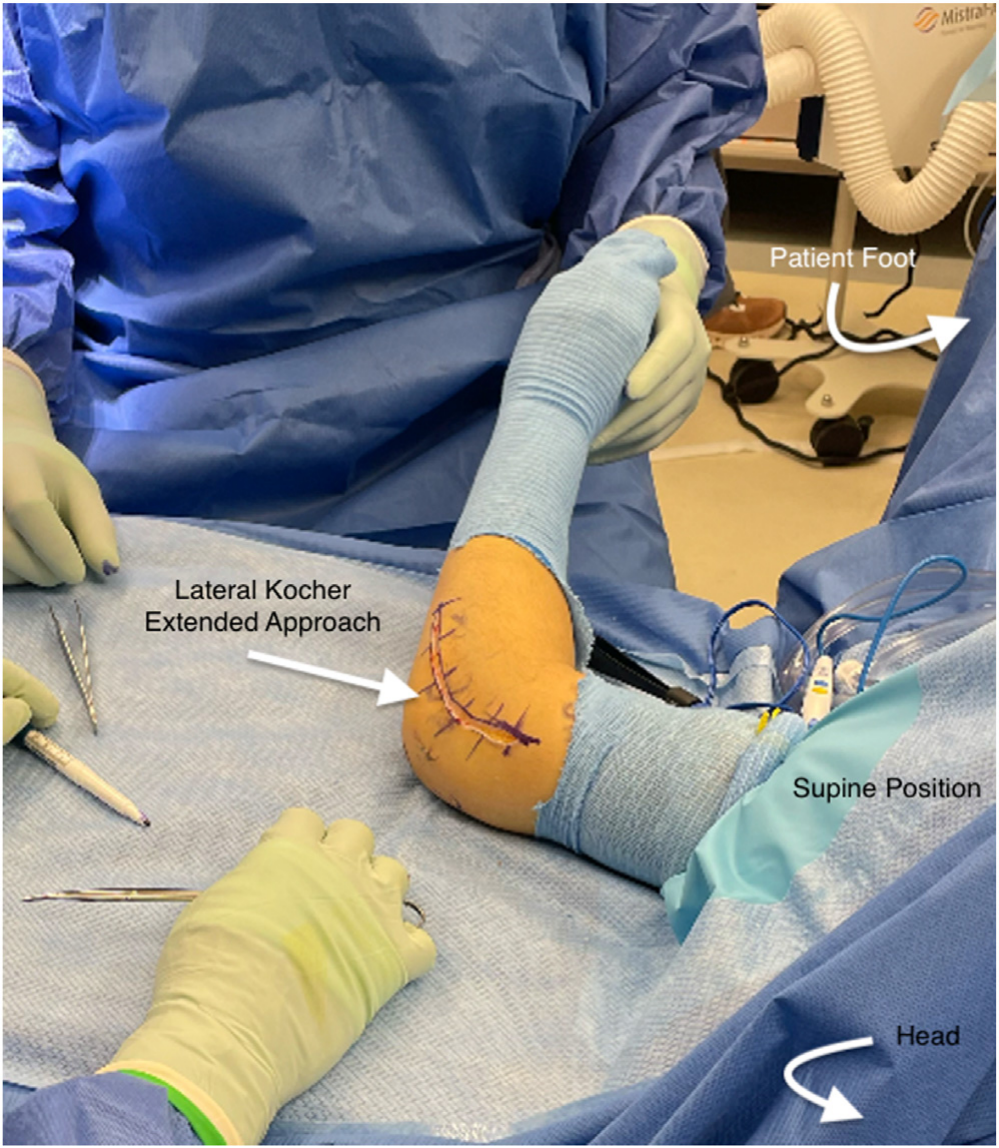
Extensile lateral approach to the left elbow. Left arm exposed in a patient lying supine.

**Fig 5:**
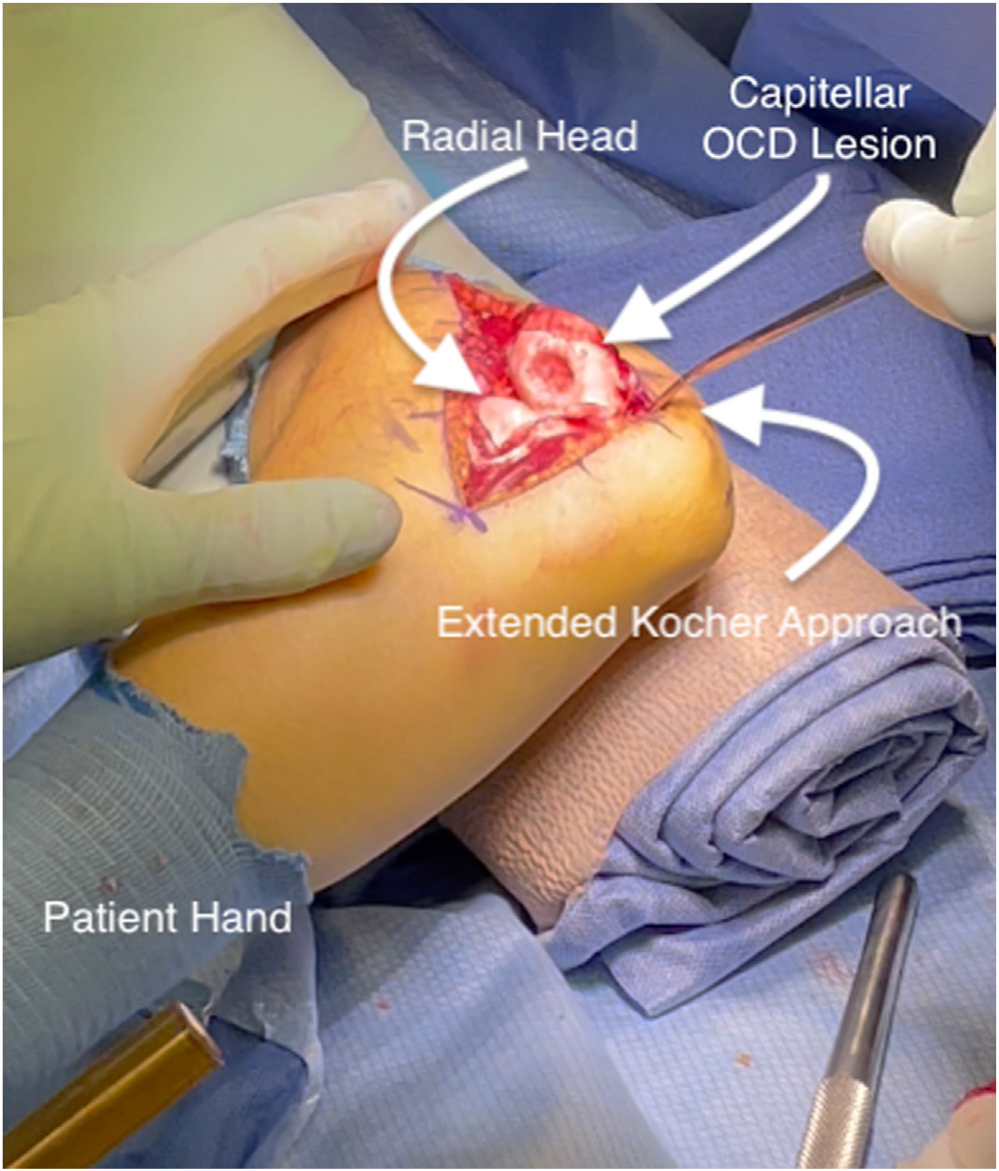
Extended lateral approach to the left elbow form the supine position showing entire LUCL complex takedown with subluxation of the ulno-humeral joint to allow complete perpendicular exposure of the capitelum. (LUCL, lateral ulnar collateral ligament; OCD, osteochondritis dissecans.)

The operative extremity is again prepped and draped in the standard sterile fashion for the second portion of the case, having clearly confirmed indications of capitellar lesions. An extensile lateral approach of approximately 10 cm is demarcated over the lateral portion of the elbow. The Kocher interval is exposed and used to enter the joint. Exposure to the level of the radial neck distally and proximally up the lateral column, taking off the lateral ulnar collateral ligament in 1 sheet, is crucial to allow unencumbered access to the capitellum (Fig 4). This allows subluxation of the radial head out of the plane of the capitellum (Fig 5). This complete visualization of the capitellum is necessary for the ability to instrument perfectly orthogonal to the capitellar surface and maximize graft placement.

### Step 4: Recipient Site Preparation

**Fig 6:**
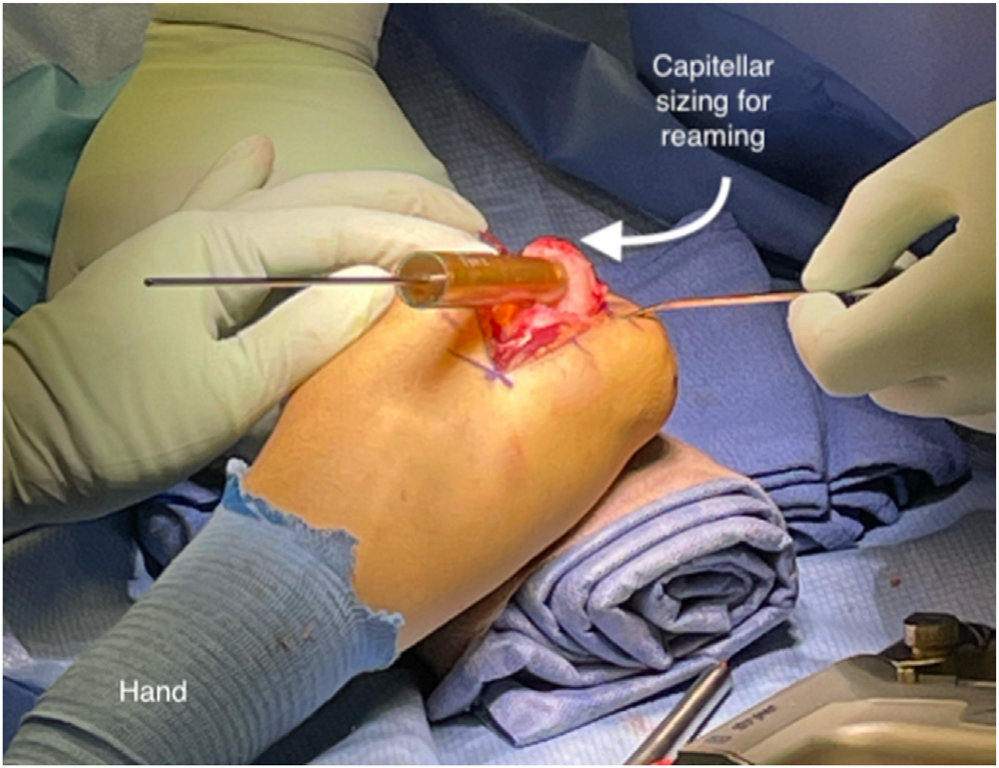
Sizing of the capitellar lesion of the left elbow.

**Fig 7:**
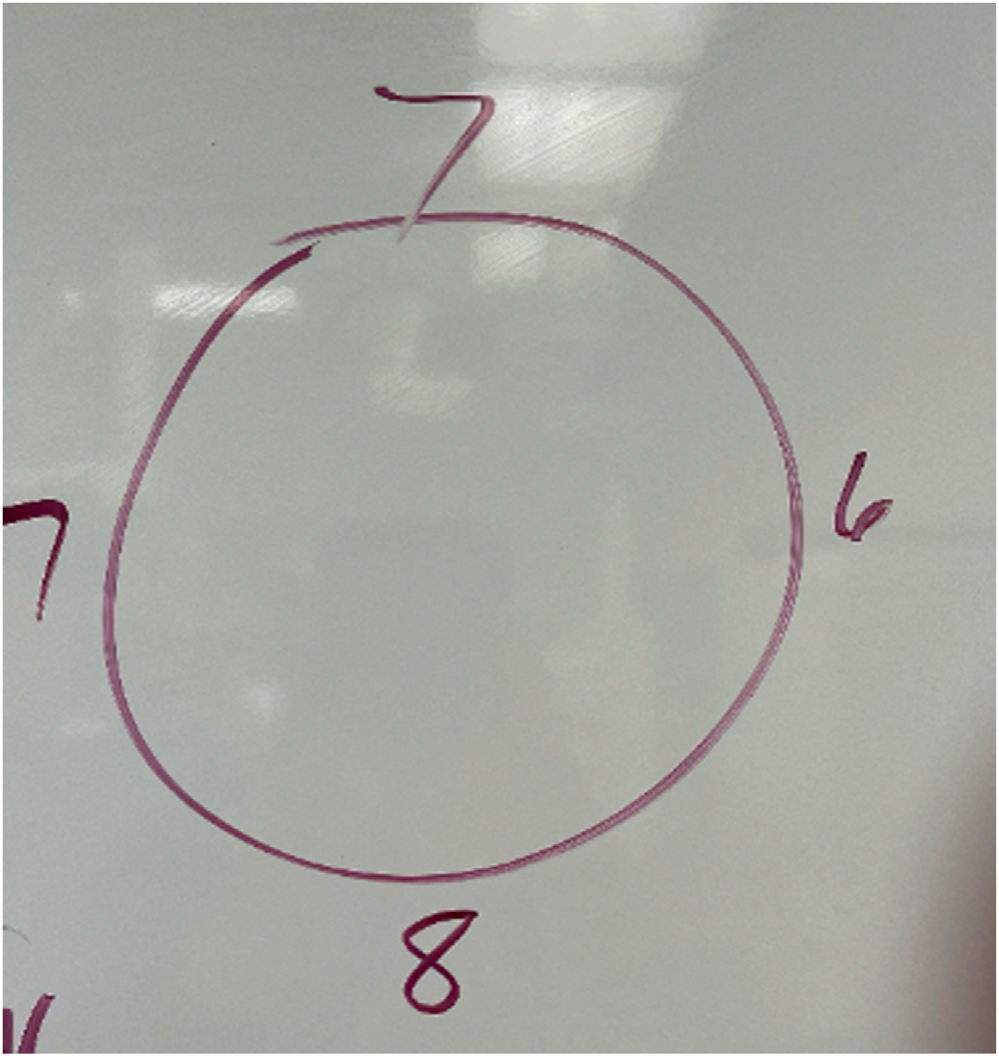
Measurement of capitellar reaming depth circumferentially.

Once exposure has been obtained, the recipient site is prepared. Borders of the lesion are clearly identified, and the mega OATS set (Arthrex) is used to find an appropriately sized preparation reamer that will remove all of the involved cartilage (Fig 6). The goal is to create a clean chondral edge with a depth of approximately 6 to 8 mm of bone that is orthogonal to the joint surface. It is crucial to place this so that the defect is contained without free edges that would compromise press fit capabilities. A K-wire is placed in the center of the lesion and reaming with the appropriately sized reamer is performed, being cautious to control thermal necrosis and maintain contained edges. The depth of the recipient site is carefully demarcated at 4 locations, and the correct orientation is maintained with the use of a surgical marker (Fig 7, Video 1).

### Step 5: Graft Preparation

**Fig 8:**
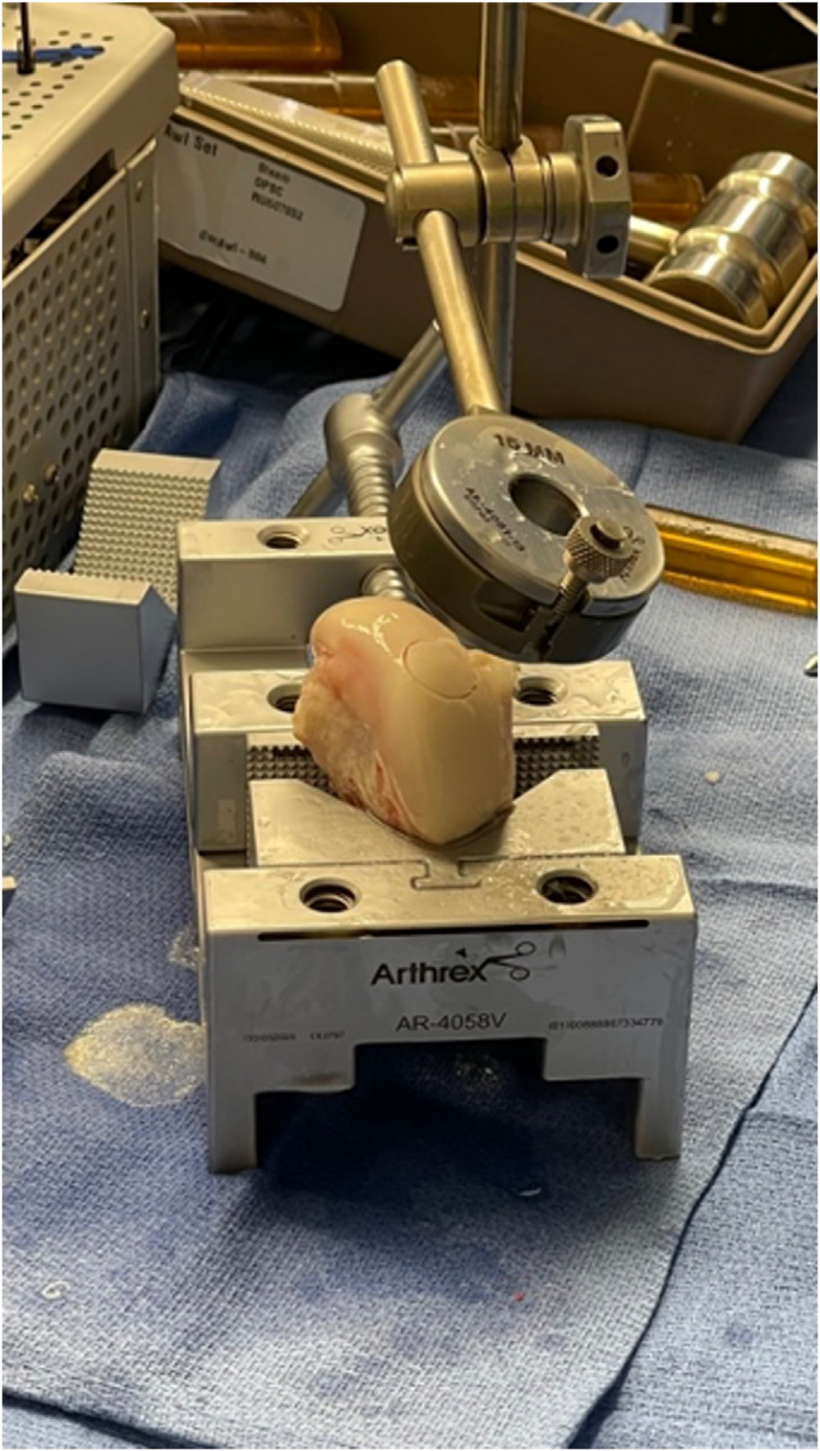
Graft table setup and preparation of a lateral femoral condyle allograft plug.

After the graft site has been prepared, a fresh whole hemicondyle of the ipsilateral distal femur is obtained. Careful selection of a surface that matches the desired contour is performed. It is crucial to assess the size and location of the recipient site to confirm both the sagittal and coronal curvatures contained in the capitellum that are required from the donor graft. A hemicondyle is positioned in a graft holder and a reciprocal reamer of the correct site is used to ream an appropriate graft. The condyle is crosscut with a sagittal saw after a surgical marker is again used to help demarcate orientation. The correct depths are then marked along the 4 recipient locations and the graft is trimmed to the correct depth. The edges of the graft are beveled to aid in placement. Finally, the graft is irrigated with pulsatile irrigation to remove all hematopoietic elements and aid in incorporation (Fig 8, Video 1).

### Step 6: Graft Placement and Closure

**Fig 9:**
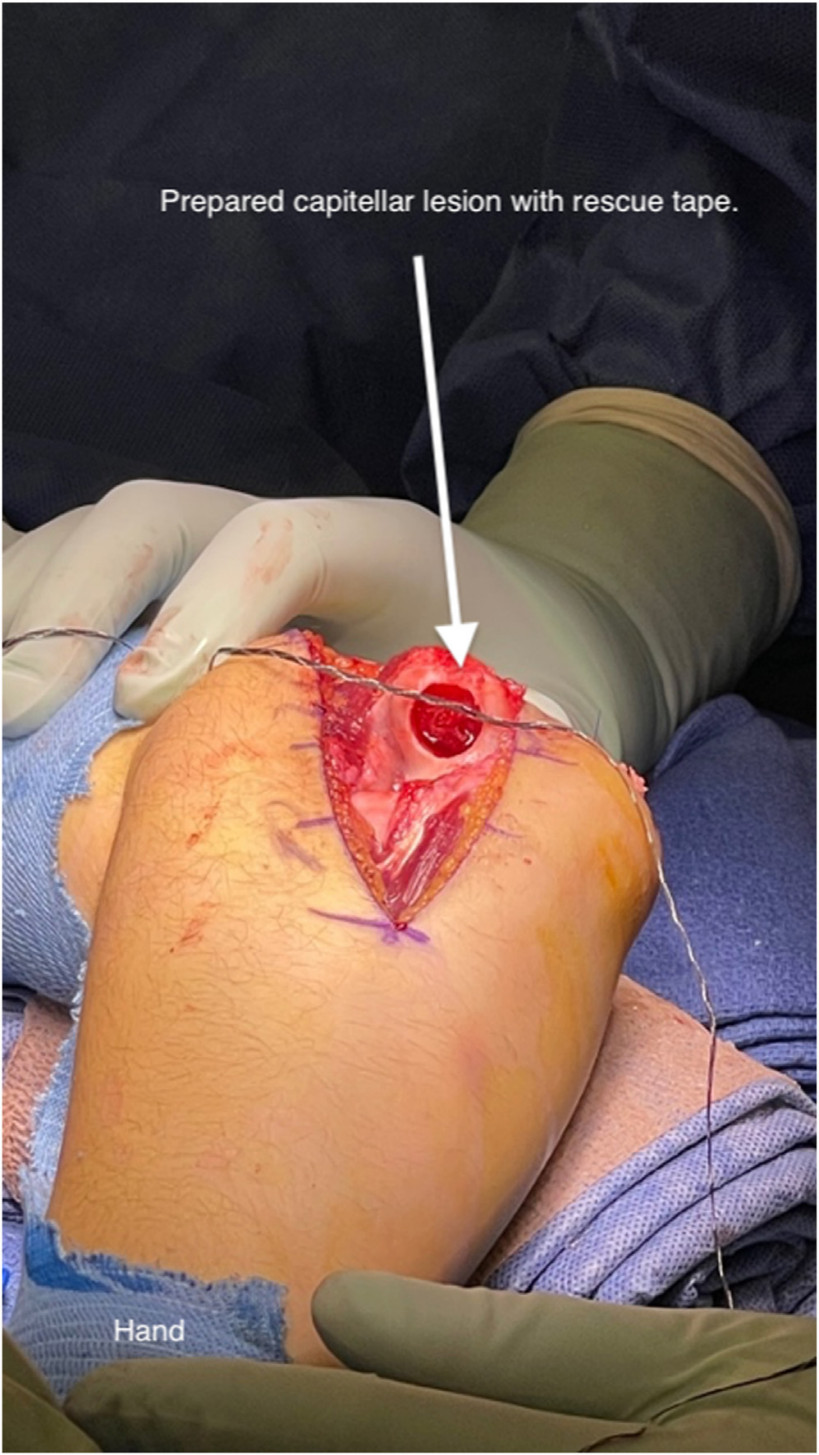
Left elbow in the supine position showing prepared capitellum with extensile exposure to allow perpendicular access to the capitellum. Safety suture placed in the base of the recipient site to allow for graft repositioning.

**Fig 10:**
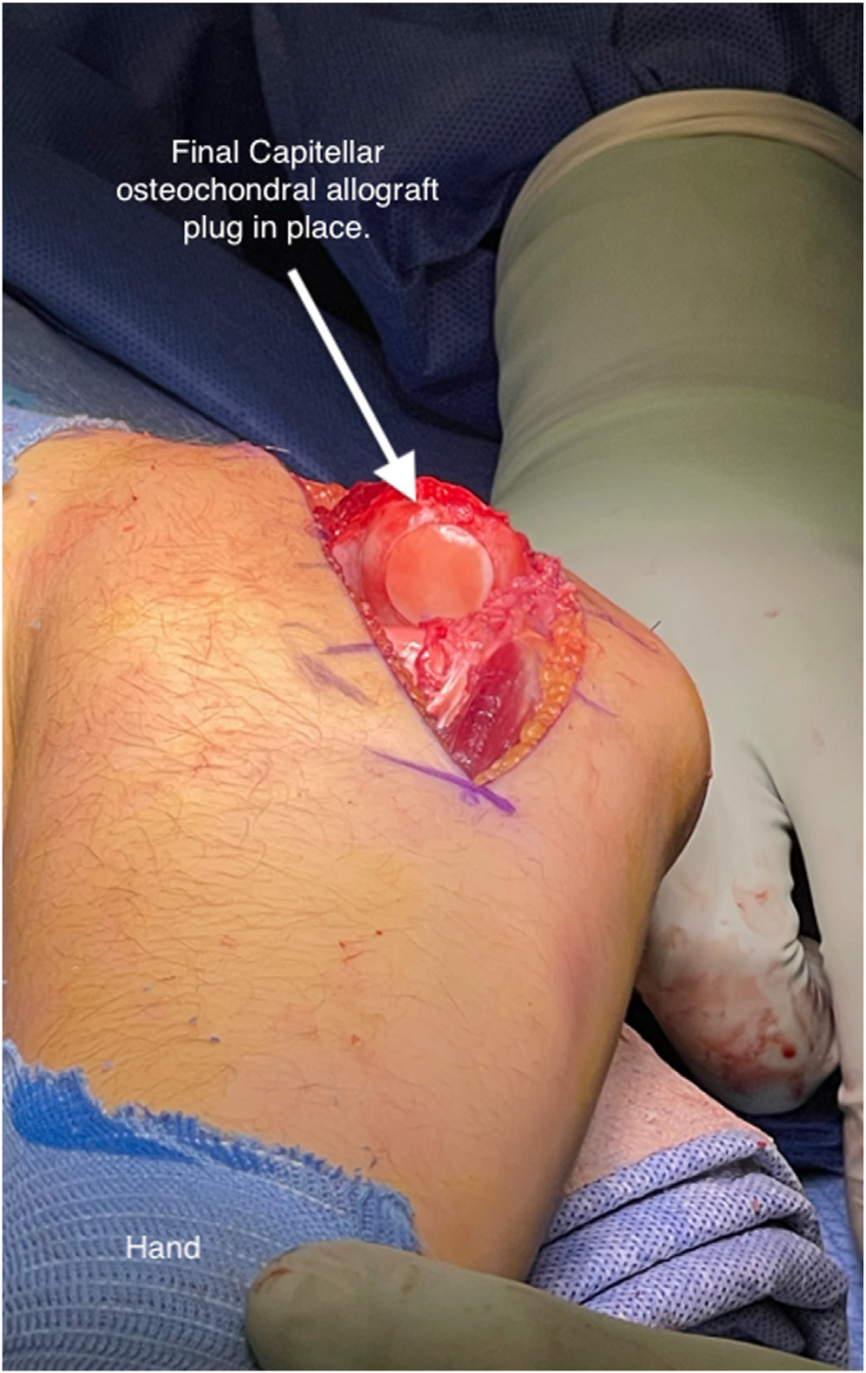
Final placement of a 15-mm osteochondral graft to left elbow capitellum visualized from an extensile lateral approach to the elbow.

A single generic tape suture is placed in the bed of the wound as a precaution (Fig 9). This allows a poorly seated or malrotated graft to be removed with gentle traction on the suture. Once in place, the graft is oriented correctly, and hand placed into the recipient site. We use thumb pressure to seat the graft. Once seated, final positioning can be aided with the use of gentle impaction. A press fit is achieved and evaluated throughout the range of motion (Fig 10). A single suture anchor (Mitek GII; DePuy Synthes) is placed in the center of the lateral epicondyle and the lateral ulnar collateral ligament is repaired back to its anatomic footprint with the remaining sheet of extensor tissue. A layered closure is performed and a long arm splint is applied (Video 1).

### Step 7: Rehabilitation Protocol

Our preferred rehabilitation protocol begins after 2 weeks of elbow splinting for soft tissue rest. At this point the patient is transitioned to a hinged brace with progressive range of motion, with the goal of full painless range of motion by the 6-week mark postoperatively. Gentle strengthening is initiated between weeks 6 and 12, with more aggressive therapy beginning at the 3-month mark. Our preferred rehabilitation program sees players start on a return to sport or throwing program at 4 to 5 months, with the goal of return to sport between 6 and 9 months.

## Discussion

OCD lesions of the elbow continue to present a diagnostic and clinical challenge in young athletes. For the rare situation in which patients fail to respond to appropriate conservative treatments, surgical options exist.^1^^,^^2^^,^^8^, ^9^, ^10^, ^11^, ^12^, ^13^ Surgical treatments in the past have included debridement, excision of loose bodies, microfracture, and osteochondral grafting.^3^^,^^4^^,^^14^^,^^15^ Autograft options for the treatment of these symptomatic lesions traditionally have included femoral trochlear autografts, lateral femoral condyle autografts, and costal osteochondral grafts.^5^^,^^11^^,^^15^, ^16^, ^17^, ^18^, ^19^

The success of autograft transplantation in these patients is well documented, with at least 2 systematic reviews showing a high rate of return to sport at or above the same level.^20^^,^^21^ Despite the success of autograft transplantation, donor site morbidity has been well documented, with some series reporting rates as high as 8%.^5^^,^^22^^,^^23^ In addition to the unavoidable presence of donor site morbidity with autograft harvest, other factors, such as small patient size, large lesion size, and preexisting patellofemoral pain, have been suggested as relative contraindications for the harvest of knee cartilage for the treatment of OCD lesions of the elbow.^4^^,^^6^^,^^7^^,^^14^ Additionally, as lesions approach larger size, morbidity increases significantly. This has driven the discussion of allograft transplantation of the capitellum, especially in cases in which more than 10-mm diameter grafts are needed. The knee literature shows excellent mid- and long-term outcomes with osteochondral allograft transplantation for even large unipolar symptomatic cartilage lesions; however, data for this in the elbow are lacking.^24^ This surgical technique shows our preferred method for allograft osteochondral transplantation from the lateral femoral condyle for the treatment of OCD of the capitellum.

This study has several limitations. Despite long-term follow up for large osteochondral allograft usage in the knee, elbow literature for large osteochondral allograft transplantation is lacking. As such, this technical description needs to be substantiated with mid- and long-term follow-up and close monitoring of patient-reported outcomes and return to sport. Additionally, cost is a major consideration for patients, and the use of non–insurance-covered allografts adds significant patient costs that need to be carefully considered.^4^ Despite these limitations, the described surgical technique illustrates the successful treatment of a large OCD lesion of the capitellum. A detailed description of the pearls and pitfalls involved in approaching such a clinically challenging entity is also provided in Table 2.

**Table 2:** Pearls and Pitfalls of Mega OCA Placement in the Capitellum

Pearls	Pitfalls
Complete removal of the attachments of the extensors and capsule off the lateral column is crucial for adequate exposure of the capitellum.	Insufficient exposure risks iatrogenic radial head damage or graft misplacement.
Fresh hemicondyle graft allows for accurate contour match with ability to harvest multiple plugs if needed.	Insufficient LUCL repair will result in iatrogenic posterolateral rotatory instability.
Multiple flexion angles of the elbow are often necessary to obtain perfect orthogonal positioning for recipient site preparation and graft placement.	

LUCL, lateral ulnar collateral ligament; OCA, osteochondral allograft.

## Disclosures

The authors declare the following financial interests/personal relationships which may be considered as potential competing interests: D.R.D. reports a consulting or advisory role with Smith & Nephew, Medical Device Business Services, DePuy Synthes, and OsteoCentric Technologies. D.R.D. also reports receiving speaking and lecture fees and travel reimbursement from Smith & Nephew.

## Supplementary Data

ICMJE author disclosure forms

ICMJE author disclosure forms

Video 1 This is a case example of the management of a large (>10 mm) osteochondritis dissecans lesion of the capitellum with an extensile approach to the left lateral elbow, lateral femoral condyle allograft preparation, and placement in the capitellum.
