# Supplementary material for: Osteochondral Allograft Transplantation to the Capitellum: Technical Considerations of a Mega Osteochondritis Dissecans Technique
Source: Arthrosc Tech. 2024 Apr 17;13(7):102997. doi: 10.1016/j.eats.2024.102997 (PMC11293318; doi:10.1016/j.eats.2024.102997)
Supplement: ICMJE author disclosure forms [file mmc1.docx]

**Declaration of interests**
 
☐ The authors declare that they have no known competing financial interests or personal relationships that could have appeared to influence the work reported in this paper.
 
☒ The authors declare the following financial interests/personal relationships which may be considered as potential competing interests:

| David Diduch reports a relationship with Smith and Nephew Inc that includes: consulting or advisory, speaking and lecture fees, and travel reimbursement. David Diduch reports a relationship with Medical Device Business Services that includes: consulting or advisory. David Diduch reports a relationship with DePuy Synthes that includes: consulting or advisory. David Diduch reports a relationship with Osteocentric Technologies that includes: consulting or advisory. If there are other authors, they declare that they have no known competing financial interests or personal relationships that could have appeared to influence the work reported in this paper. |
| --- |
